# Inherited Prion Disease A117V Is Not Simply a Proteinopathy but Produces Prions Transmissible to Transgenic Mice Expressing Homologous Prion Protein

**DOI:** 10.1371/journal.ppat.1003643

**Published:** 2013-09-26

**Authors:** Emmanuel A. Asante, Jacqueline M. Linehan, Michelle Smidak, Andrew Tomlinson, Andrew Grimshaw, Asif Jeelani, Tatiana Jakubcova, Shyma Hamdan, Caroline Powell, Sebastian Brandner, Jonathan D. F. Wadsworth, John Collinge

**Affiliations:** Medical Research Council Prion Unit and Department of Neurodegenerative Disease, University College London Institute of Neurology, Queen Square, London, United Kingdom; University of Alberta, Canada

## Abstract

Prions are infectious agents causing fatal neurodegenerative diseases of humans and animals. In humans, these have sporadic, acquired and inherited aetiologies. The inherited prion diseases are caused by one of over 30 coding mutations in the human prion protein (PrP) gene (*PRNP*) and many of these generate infectious prions as evidenced by their experimental transmissibility by inoculation to laboratory animals. However, some, and in particular an extensively studied type of Gerstmann-Sträussler-Scheinker syndrome (GSS) caused by a *PRNP* A117V mutation, are thought not to generate infectious prions and instead constitute prion proteinopathies with a quite distinct pathogenetic mechanism. Multiple attempts to transmit A117V GSS have been unsuccessful and typical protease-resistant PrP (PrP^Sc^), pathognomonic of prion disease, is not detected in brain. Pathogenesis is instead attributed to production of an aberrant topological form of PrP, C-terminal transmembrane PrP (^Ctm^PrP). Barriers to transmission of prion strains from one species to another appear to relate to structural compatibility of PrP in host and inoculum and we have therefore produced transgenic mice expressing human 117V PrP. We found that brain tissue from GSS A117V patients did transmit disease to these mice and both the neuropathological features of prion disease and presence of PrP^Sc^ was demonstrated in the brains of recipient transgenic mice. This PrP^Sc^ rapidly degraded during laboratory analysis, suggesting that the difficulty in its detection in patients with GSS A117V could relate to post-mortem proteolysis. We conclude that GSS A117V is indeed a prion disease although the relative contributions of ^Ctm^PrP and prion propagation in neurodegeneration and their pathogenetic interaction remains to be established.

## Author Summary

Prions are infectious agents causing incurable brain disease in humans and animals. Prion diseases are by definition transmissible, which means that it should be possible to experimentally transfer disease from patient brain tissue to laboratory animals by inoculation. While many forms of prion disease have been shown to be experimentally transmissible, some inherited forms, in particular, Gerstmann-Sträussler-Scheinker syndrome (GSS) associated with the substitution of valine for alanine at amino acid position 117 (GSS A117V) of the human prion protein gene have not. This has led to the suggestion that such syndromes are not true prion diseases and are better designated non-transmissible proteinopathies. Since prions may transmit more efficiently when the host's normal prion protein amino acid sequence matches that of the infecting prion, we generated transgenic mice expressing human prion protein with the same amino acid sequence found in A117V GSS. We found that brain tissue from GSS A117V patients could transmit disease to these mice, producing the typical brain lesions associated with GSS A117V. We therefore conclude that GSS A117V is an authentic prion disease.

## Introduction

According to the widely accepted “protein-only” hypothesis [1], an abnormal isoform (PrP^Sc^) of host-encoded cellular prion protein (PrP^C^) is the principal, and possibly the sole, constituent of the transmissible agent or prion [2]. Prions exist in multiple strains which are thought to represent distinct polymeric forms of misfolded PrP which faithfully propagate by recruitment of host PrP^C^ onto pre-existing seeds or fibrils (for review see [3]). Human prion diseases may occur sporadically, be acquired by infection with environmental prions, or be inherited as autosomal dominant conditions as a result of one of more than 30 different coding mutations in the human PrP gene (*PRNP*) [4]. The cause of neuronal dysfunction and death in prion disease is unclear but neurotoxicity may be uncoupled from infectivity suggesting that prions themselves may not be directly neurotoxic and other PrP species might be involved in mediating toxicity [3], [5], [6].

By definition, prion diseases are transmissible, and while all the sporadic and acquired human prion diseases have been transmitted to laboratory animals, not all of the inherited forms have. It has been suggested therefore that some of these inherited neurodegenerative syndromes are prion proteinopathies with a distinct pathogenesis that may not involve production of infectious prions. One inherited prion disease (IPD) in particular, associated with an alanine to valine substitution at residue 117 of PrP (A117V), has been proposed to cause neurodegeneration in the absence of PrP^Sc^, with pathogenesis mediated by aberrant production of ^Ctm^PrP, a transmembrane form of PrP [7]. It has also been proposed that PrP^Sc^ accumulation in other forms of prion disease may cause pathology by inducing the synthesis of ^Ctm^PrP *de novo* [8]. This aberrant topologic form of PrP has been hypothesised to cause neurologic dysfunction by disrupting the function of mahogunin, a cytosolic ubiquitin ligase whose loss causes spongiform neurodegeneration [9]. However, a recent study using mice lacking the Mahogunin Ring Finger 1 (MGRN1) E3 ubiquitin ligase concluded that disruption of MGRN1-dependent pathways does not play a significant role in the pathogenesis of prion diseases [10].

A117V is one of the IPD mutations associated phenotypically with the Gerstmann-Sträussler-Scheinker syndrome (GSS) which usually presents clinically as a progressive cerebellar ataxia with dementia occurring later in a clinical course usually far more protracted than that of Creutzfeldt-Jakob disease (CJD) [11]. Pathologically, GSS is characterised by the presence of multicentric PrP amyloid plaques. However, in common with other IPD's, A117V has a wide phenotypic diversity at both the clinical and pathological level even within the same kindred [11]. This disease was originally misdiagnosed as Alzheimer's disease [12] before the advent of PrP immunohistochemistry [13] and the subsequent identification of the A117V mutation by *PRNP* sequencing [14].

A major determinant of phenotypic heterogeneity in prion diseases of humans and animals is prion strain diversity, with distinct prion strains producing characteristic clinical and pathological phenotypes [15]. Prion strains can be distinguished by biochemical differences in PrP^Sc^, referred to as molecular strain typing [16]. In a number of inherited prion diseases, distinct PrP^Sc^ types have been reported associated with the same *PRNP* pathogenic mutation and this may in part explain phenotypic heterogeneity (for review see [17], [18]). In this regard while classical CJD is typically characterised by proteinase K-resistant PrP fragments of ∼21–30 kDa on immunoblots [19] most GSS cases show additional low molecular mass fragments of 7–15 kDa [19]–[27]. Notably, the major protease-resistant peptide extracted from brains of GSS A117V patients is a ∼7–8 kDa PrP fragment [26], and to-date it has not been possible to detect proteolytic fragments of molecular mass 21–30 kDa in these samples. The pathogenic role of the PrP species from which the 8 kDa fragment is generated is not clear because, inocula containing this fragment induced conversion of murine 101L-PrP into amyloid but did not induce spongiform neurodegeneration in the recipient mouse brains [28]. These facts coupled with the negative experimental transmission data have led to the suggestion that GSS A117V may not be an authentic prion disease and would be more accurately described as a non-transmissible proteinopathy [29], [30].

That most sporadic and acquired CJD occurred in individuals homozygous at *PRNP* polymorphic codon 129 supported the view that prion propagation proceeded most efficiently when the interacting PrP^Sc^ and PrP^C^ were of identical primary structure [31], [32]. It has been demonstrated that the species barrier may be abrogated in transgenic mice expressing PrP homologous to that of the exogenous PrP^Sc^ [33]. This is also the case with transmission of human prion diseases. Classical CJD transmits rarely if at all to wild type mice but highly efficiently (indeed without a transmission barrier) to mice expressing human (and not mouse) PrP [34], [35]. However, prion strain type may also play a key role in transmission barriers, which are thought to be mediated via conformational selection where a given PrP primary structure has a preferred subset of disease-associated conformations it can adopt [3], [36]. While therefore it is possible that some naturally occurring human prion strains could transmit more efficiently for example to wild type mice rather than to mice transgenic for a particular human PrP polymorph (as is the case for vCJD for example [37]), it is logical to test for transmissibility of GSS A117V using transgenic mice expressing only human PrP 117V.

Here we present the first evidence that IPD A117V cases produce transmissible prions; previous transmission attempts may have failed from use of inappropriate experimental models. Furthermore, we show that the previous failure to detect PrP^Sc^ in GSS A117V patient brain may have been due to its unusual instability with consequent loss by post-mortem proteolysis in human brain samples.

## Results

### Susceptibility of transgenic mice expressing only human PrP 117V to sporadic and acquired CJD prions

We produced transgenic mice homozygous for both a human PrP 117V, 129V transgene array and murine PrP null [38] alleles (*Prnp^o/o^*), designated Tg(HuPrP^117V,129V+/+^ *Prnp^o/o^*)-31 (hereafter referred to as 117VV Tg31), with human PrP expression levels three times that of pooled normal human brain (data not shown). We studied an ageing cohort of 20 mice for evidence of spontaneous neurodegeneration, however all of these uninoculated mice died of intercurrent illnesses or old age between 460 and 904 days without developing neurological disease. In addition, three out of a further control group of five mice mock-inoculated with PBS buffer lived to between 344 and 735 days post-inoculation without developing neurological signs. One mouse was scored clinically sick at 303 days post inoculation (Table 1) but this and two other PBS-inoculated mice had no evidence for pathological PrP in brain by either immunoblotting or immunohistochemistry (IHC). One mouse, culled at 582 days post-inoculation due to intercurrent illness, although negative for PrP^Sc^ by immunoblotting was found to have minor PrP immunoreactivity in the anterior commissure by IHC (Figure S1A). This finding in a single sample was not studied further.

**Table 1:** Primary transmission of classical CJD, GSSA117V and vCJD prions to transgenic mice expressing huPrP^117V,129V+/+^ (117VV Tg31).

	Inoculum								
Aetiology	Code	*PRNP* 129 genotype	Human PrP^Sc^ type*	Clinical signs	Incubation period (days ± sem)	Positive by IHC	Positive by IB		Total affected†
							8 kDa	21–30 kDa	
PBS	I056	-	-	1/4	303	1/4	0/4	0/4	1/4‡
Sporadic CJD	I022	VV	T2	2/6	263, 303	5/6	0/4	3/4	5/6
	I1478	MV	T2	0/6	>320	1/5	0/2	0/2	1/6
Iatrogenic CJD	I026 (DM)	MM	T2	0/5	>312	0/2	ND	ND	0/5
	I1477 (GH)	MV	T3	0/6	>247	0/6§	0/6	1/6	1/6
	I2651 (GH)	VV	T3	1/6	748	1/6	0/6	0/6	1/6
129 VV Tg152-passaged vCJD	I785	VV	T5	0/6	>558	0/5	ND	ND	0/6
vCJD	I336	MM	T4	0/7¥	>291	0/7	0/7	0/7	0/7
IPD A117V	I514	VV	ND	2/9	609, 673	6/8	4/8	4/8	8/9
	I1321	VV	ND	1/5	634	1/4	1/5	1/5	1/5
	I1322	VV	ND	2/6	634, 634	2/2	4/4	0/4	6/6

IPD = inherited prion disease; IHC = immunohistochemistry; IB = immunoblotting; ND = not determined; GH = growth hormone; DM = dura mater.

* According to classification of Hill *et al*. [53].

† Positive either by clinical signs, western blotting and/or immunohistochemistry; primary antibody was either 3F4 or ICSM 35.

‡ One PBS-inoculated mouse culled at 582 days post-inoculation due to intercurrent illness, had minimal PrP plaques (Figure S1A) in the anterior commissure but was negative by IB.

§ One mouse was positive by the presence of PrP21–30 kDa on immunoblot (Figure 3B, lane 4) but tissue was unavailable for immunohistochemical analysis.

¥ Post-inoculation survival period (days): 292, 387, 450, 455, 498, 515 and 547.

To assess the susceptibility of these novel transgenic lines to prion infection, we first inoculated them with well characterised isolates of classical CJD with proven transmissibility to mice expressing wild-type human PrP [35], [37], [39], [40] although recognising that the presence of the A117V mutation may introduce a transmission barrier to prions generated from wild-type PrP. Sporadic CJD isolate I022 (*PRNP* 129VV with type 2 PrP^Sc^) caused clinical disease in 2/6 mice with relatively short incubation periods of 263 and 303 days post-inoculation (Table 1). Although clinical attack rate was low, most mice (5/6) were subclinically infected and showed positivity for abnormal PrP by IHC and/or immunoblotting (Table 1). In contrast, other inocula comprising sporadic CJD and iatrogenic CJD with different *PRNP* codon 129 status and PrP^Sc^ types, and also vCJD and vCJD passaged in 129VV Tg152 mice (which contained type 5 PrP^Sc^ [37], [40]) transmitted very poorly or not at all (Table 1). Collectively these findings show that at 3-fold expression level of human PrP, 117VV Tg31 mice can replicate human prions, although this varies with prion strain and codon 129 genotype effects.

### Susceptibility of transgenic mice expressing only human PrP 117V to IPD A117V inocula

117VV Tg31 mice were inoculated with three isolates of GSS A117V and remarkably all resulted in transmission with clinically affected mice (Table 1). To our knowledge this is the first time these isolates have been shown to have transmissible prions. However, it should be noted that clinical transmissions were associated with extremely long incubation periods, ranging from 609 to 673 days post-inoculation (Table 1). It is therefore unsurprising that previous attempts to transmit this disease, into animals expressing endogenous levels of a PrP of different primary structure, were completely unsuccessful [41], [42].

### Sub-clinical prion infection in mice expressing human PrP A117V

**Figure 1:**
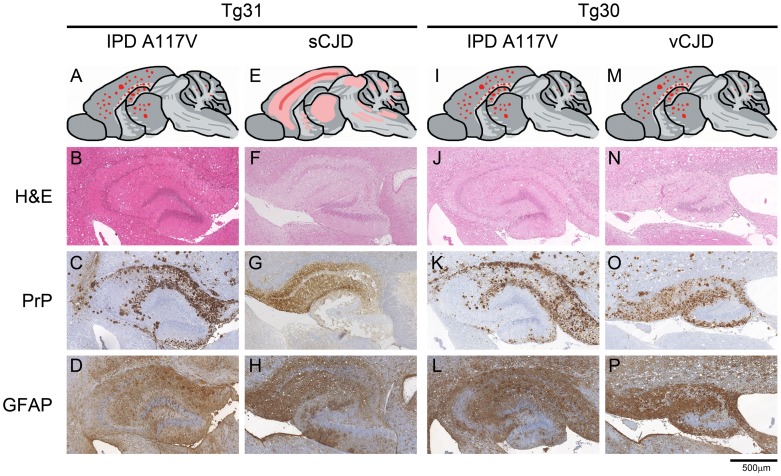
Neuropathological analysis of transgenic mouse brain. Panels A, E, I and M, schematics showing regional distribution of abnormal PrP deposits. Note that these panels reflect the overall spatial distribution of neuropathology and are not meant to indicate precise representations of individual brains. Panels B, F, J and N, H&E staining demonstrating spongiform neurodegeneration in the hippocampal areas. Panels C, G, K and O, PrP immunohistochemistry using anti-PrP monoclonal antibody ICSM 35 demonstrates abnormal PrP immunoreactivity. Panels D, H, L and P, GFAP staining demonstrating gliosis in the hippocampal areas. A–D, IPD A117V prions inoculated to 117VV Tg31 mouse; E–H, sporadic CJD prions inoculated to 117VV Tg31 mouse showing distinctive diffuse synaptic PrP deposition characteristic of sCJD prions (panel G); I–L, IPD A117V prions inoculated to 117VV Tg30 mouse. M–P, vCJD prions inoculated to 117VV Tg30 mouse showing abundant non-florid PrP plaques (panel O). Scale bar = 500 µm for all panels except A, E, I and M.

**Figure 2:**
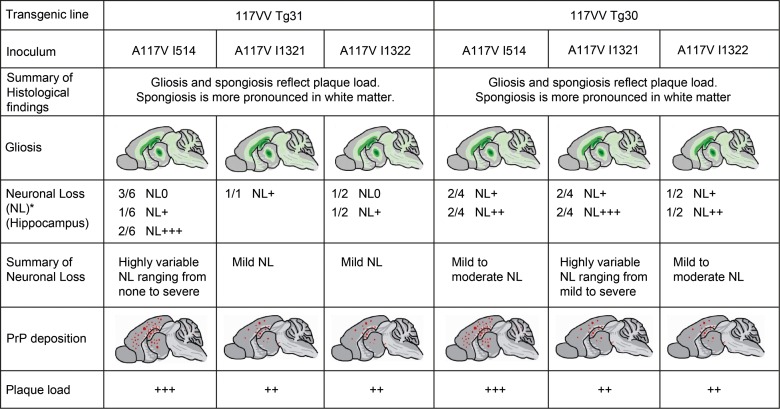
Overview of histological findings in 117VV HuPrP transgenic mice challenged with A117V prion isolates. The figures are schematic drawings reflecting the overall spatial distribution and intensity pattern of the gliosis or PrP deposition within the experimental groups. They are not meant to indicate precise representations of individual brains. * Definition of values for neuronal loss: NL0: No neuronal loss; NL+: Drop out of single neurones either focally or within the Ammon's horn (AH), leaving the AH continuity intact; NL++: Focal or regional drop out, interrupting the continuity of the AH and creating a small-medium gap (up to 1/3 of the length of the AH); NL+++: Neuronal drop out leaving gaps of more than 1/3 of the AH's length. Ratios represent the proportion of samples with the corresponding neuronal loss score.

We investigated all the clinically unaffected mice challenged with brain homogenates from GSS A117V patients or classical CJD prions for evidence of subclinical infection [5], [39], [43] by both PrP immunohistochemistry and immunoblotting. We found that 6/8 of the mice inoculated with GSS A117V prion isolate I514 were positive by immunohistochemistry (Figure 1 A–D), although only 2/9 showed clinical signs (Table 1). In contrast to sporadic CJD inoculum I022 which produced only synaptic type PrP deposits (Figure 1E and G), all three IPD A117V inocula resulted in intense deposition of PrP plaques in cerebral cortex, hippocampus, thalamus and cerebellum (Figure 1A and C). There was neuronal loss (Figure S1B and Figure 2) and spongiosis, more pronounced in the white matter (Figure 1B), and gliosis (Figure 1D and Figure 2) that reflected the extent of the PrP plaque load. Sub-clinical infection was also prominent in 117VV Tg31 mice challenged with sporadic CJD inoculum I022, with 5/6 mice being positive by immunohistochemical analysis (Table 1, Figure 1 E–H) despite a low clinical attack rate of 2/6.

### PrP^Sc^ can be propagated in mice expressing only human PrP 117V

The classical proteolytic PrP^Sc^ fragments of ∼21–30 kDa have to-date not been detected in brain from A117V patients. We analysed the brains of all clinically affected mice and those that died of inter-current illnesses by immunoblotting for the presence of PrP^Sc^. We first confirmed that 117VV Tg31 mice are capable of producing stable PrP^Sc^ by analysing brains of mice inoculated with sporadic CJD prions. In order to adequately digest PrP^C^ in these mice, we used stringent PK digestion conditions of 100 µg/ml incubated at 37°C for 1 hour, and demonstrated the presence of PrP^Sc^ in the brains of A117V Tg31 mice inoculated with sporadic CJD inoculum.

**Figure 3:**
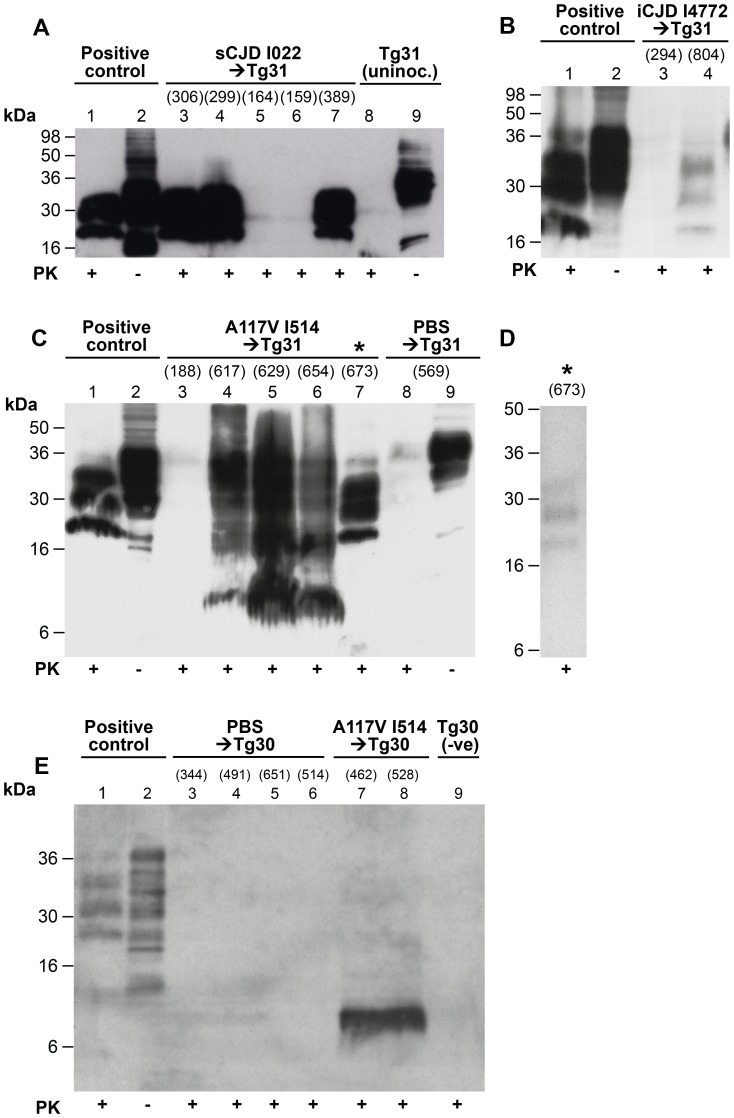
Immunoblot analysis of abnormal PrP propagated in the brains of 117VV transgenic mice challenged with IPD A117V and classical CJD. Mice were inoculated with classical CJD and GSS A117V brain. Immunoblots were analysed by enhanced chemiluminescence with monoclonal anti-PrP antibody ICSM 35. The numbers in parentheses above relevant lanes, represent the number of days each mouse survived post-inoculation. The provenance of each brain sample is designated above each lane. (A) Immunoblots of sporadic CJD-inoculated 117VV Tg31 mice (lanes 3–7) showing PrP^Sc^ resistant to harsh proteinase K (PK) digestion performed with 100 µg/ml PK at 37°C for 1 h (lanes 3, 4, and 7). Positive control was from a transgenic mouse expressing wild type HuPrP-129MV challenged with the same CJD inoculum (lanes 1and 2). An uninoculated 117VV Tg31 mouse brain is shown in lanes 8 and 9. (B) Brain homogenate of a 117VV Tg31 mouse inoculated with iatrogenic CJD prions that died without clinical disease at 804 days post-infection, showing weakly detectable PrP^Sc^ partially resistant to harsh PK digestion of 100 µg/ml for 1 hour at 37°C (lane 4) compared to the same control as in Figure 3A (lanes 1 and 2). Brain homogenate of a mouse killed relatively early at 294 days post-inoculation, shows no detectable PrP^Sc^ (lane 3). (C) Immunoblots of brains of five separate 117VV Tg31 mice all inoculated with the same GSS A117V patient brain homogenate showing the presence of PrP^C^, PrP^Sc^ and 8 kDa PrP fragment following harsh PK digestion at 100 µg/ml PK at 37°C for 1 hour (lanes 3–7). Under these conditions PBS-inoculated age-matched control 117VV Tg31 mouse brain shows only residual PrP^C^ signal on long exposure (lane 8). Brain homogenate of a mouse culled relatively early at 188 days post-inoculation, compared to the group mean survival post-inoculation of >616 days, showed no detectable PrP^Sc^ (lane 3). One 117VV Tg31 mouse was clinically sick at 673 days post-infection, and its brain sample shows complete digestion of PrP^C^ and the presence of classical PrP^Sc^ (lane 7, denoted by *) confirming adequacy of the PK digestion conditions. (D) Immunoblotting was repeated for all samples shown in lanes 4–7 of Figure 3C. These samples had undergone only one further freeze–thaw cycle before PK digestion. Compared with the readily detectable abnormal PrP signals seen in Figure 3C, only one sample (denoted by *) now showed the presence of classical PrP^Sc^ but at reduced signal strength, and only after using PK at a reduced concentration of 10 µg/ml. In other samples, only an 8 kDa PrP fragment could be variably detected but after using reduced PK concentrations (see Figure S2B lanes 3 and 4). (E) Immunoblot showing only the 8 kDa PrP fragment associated with A117V-challenged 117VV Tg30 mouse brains analysed with 50 µg/ml PK at 37°C for 1 hour (lanes 7 and 8), whereas PrP in brain homogenates of PBS-challenged Tg30 mice (lanes 3–6) and uninoculated age-matched Tg30 mouse brain (lane 9) is completely digested under the same conditions. Positive control in lanes 1 and 2 is brain homogenate of a transgenic mouse expressing wild type HuPrP (129MM Tg35) that was challenged with classical CJD.

Immunoblots show clear evidence that 117V PrP^C^ is convertible to PrP^Sc^ in 117VV Tg31 mice challenged with sporadic CJD inoculum I022 (Figure 3A, lanes 3, 4 and 7) and is present at similar levels in control mice expressing wild type human PrP-129MV challenged with the same prion inoculum (Figure 3A, lane 1). Transmission of iatrogenic CJD prion isolate I1477 to 117VV Tg31 mice shows a low intensity positive signal associated with the brain of a subclinically infected mouse culled 804 days post-inoculation (Figure 3B lane 4), which when compared with the absence of signal in a second mouse culled relatively early at 294 days post-infection (lane 3) probably reflects the relative abundance of 117V PrP^Sc^ accumulated over the respective survival periods.

Having established that 117V PrP^C^ would support the propagation of conventional PrP^Sc^ in our transgenic mice, we analysed brains of 117VV Tg31 mice that were inoculated with GSS A117V prions for the presence of disease-related PrP. Encouraged by the confirmation that these 117VV mice can replicate human prions, and to adequately digest PrP^C^ in these mice which express at higher levels, we used relatively harsh PK conditions (100 µg/ml PK at 37°C for 1 hour) and found that five brain samples analysed showed variable PK resistance (Figure 3C, lanes 3–7). Brain samples appear to have achieved only partial digestion even under these conditions, and displayed concurrently the presence of PrP^C^, PrP^Sc^ 21–30 kDa fragments and extra fragments of about 7–8 kDa (Figure 3C lanes 4–6). The presence of multiple PK digestion products seen on immunoblots was not due to inadequate PK digestion parameters because under the same conditions an inoculated 117VV Tg31 mouse that was killed due to intercurrent illness at 188 days post-inoculation (Figure 3C, lane 3), and a PBS-inoculated control mouse killed due to intercurrent illness at 569 days post-inoculation (Figure 3C, lane 8) showed only residual PrP^C^ signal that was only visible after long exposure.

Notably, one brain sample (Figure 3C, lane 7) achieved complete digestion with 100 µg/ml PK at 37°C for 1 hour, and clearly shows the presence of PrP^Sc^ at a level comparable to the positive control sample in lane 1. Interestingly, the 8 kDa PrP fragment was not detected in this sample.

### 117V- human PrP^Sc^ is more labile than classical CJD PrP^Sc^

As this was the first demonstration of detectable classical PrP^Sc^ (generating PK-resistant fragments equivalent to PrP^27–30^ [44]) associated with GSS A117V, we sought to reproduce the immunoblotting results but we were surprised to find that after freeze-thawing of the brain homogenates, we were unable to demonstrate PrP^Sc^ under the same harsh proteinase K (PK) conditions of 100 µg/ml at 37°C for 1 hour (Figure S2 A and B). Figure 3D shows the same sample in Figure 3C lane 7 that on repeat western blotting and exposure for the same length of time showed only a weak PrP^27–30^ signal at much reduced PK concentration of 10 µg/ml digested at 37°C for 1 hour. Repeat immunoblotting of the three other 117VV Tg31 brain homogenates shown in Figure 3C lanes 4–6, even at drastically reduced PK concentrations were negative for disease-associated PrP bands and showed only residual non-digested bands corresponding to PrP^C^ (data not shown). Of note, immunoblotting of the same samples in the absence of PK digestion showed that PrP^C^ remained relatively stable in these samples (data not shown). These results strongly suggest that 117V PrP^Sc^ is significantly more labile than that seen in CJD and other human prion diseases. The remarkable difference in migration patterns between classical CJD-challenged (Figure 3A lanes 3, 4, and7) and those of IPD A117V-challenged Tg31 mice (Figure 3C lanes 4–5) is a further reflection of the unique properties of A117V prions that set them apart from those of classical CJD prions.

### Transmission of human cases to a further HuPrP 117V-expressing transgenic line

To corroborate these novel findings, we also inoculated a second transgenic line expressing HuPrP 117V PrP^C^, called Tg(HuPrP^117V,129V+/+^ *Prnp^o/o^*)-30 (designated 117VV Tg30), with the same three IPD A117V prion isolates in addition to one case of classical CJD and the same case of vCJD (Table 2). The 117VV Tg30 mice were produced similarly to 117VV Tg31 mice but have a level of human PrP expression two-fold higher than a pooled normal human brain standard (data not shown), as compared with the 3-fold PrP overexpression in the 117VV Tg31 line. Consistent with the low rate of clinical disease in 117VV Tg31 mice, the 117VV Tg30 mice did not show a single case of clinical disease from any of the inocula administered (Table 2). However, as seen with prion-inoculated 117VV Tg31 mice, evidence of sub-clinical prion infection as measured by positive immunohistochemistry was seen in the majority of inoculated mice (Table 2 and Figure 1, I–L). Additionally, immunohistochemical analysis of the brains of 117VV Tg30 mice inoculated with GSS A117V prion isolate I514 all showed pathological lesions characterised by gliosis (Figure 1L) and spongiosis (Figure 1J) that reflected the level of PrP plaques (Figure 1K) deposited in a similar pattern to 117VV Tg31 mice described above. Spongiosis was more pronounced in white matter and neuronal loss was prominent (Figure S1B and Figure 2). Two other A117V prion inocula (I1321 and I1322) produced neuropathologically similar patterns to that of I514, though the plaque load was slightly less (Figure 2). In all GSS A117V prion-infected 117VV Tg30 mice only the 8 kDa PrP fragment was detected (Figure 3E lanes 7 and 8).

**Table 2:** Primary transmission of classical CJD, Inherited Prion Disease A117V and vCJD prions to transgenic mice expressing huPrP^117V,129V+/+^ (117VV Tg30).

	Inoculum								
Aetiology	Code	*PRNP* 129 genotype	Human PrP^Sc^ type*	Clinical Signs	Incubation period (days ± sem)	Positive by IHC	Positive by IB		Total affected†
							8 kDa	21–30 kDa	
PBS	I056	-	-	0/4	>490	0/4	0/4	0/4	0/4
IPD A117V	I514	VV	N/D	0/5	>400	4/4	5/5	0/5	5/5
	I1321	VV	N/D	0/5	>461	4/5	5/5	0/5	5/5
	I1322	VV	N/D	0/4	>327	3/3	3/4	0/4	4/4
Iatrogenic (GH)	I1263	VV	T3	0/4	>350	1/2	ND	ND	1/4
vCJD	I336	MM	T4	0/7‡	>387	3/4	5/7	2/7	5/7

IPD = inherited prion disease; IHC = immunohistochemistry; IB = immunoblotting; ND = not determined; GH = growth hormone.

* According to classification of Hill *et al*. [53].

† Positive either by clinical signs, western blotting and/or immunohistochemistry; primary antibody was either 3F4 or ICSM 35.

‡ Post-inoculation survival period (days): 388, 650, 627, 694,727, 799 and 811.

Interestingly, and in contrast to the Tg31 mice with higher levels of expression of the mutant protein, we observed spontaneous clinical disease in three mice at between 476 and 742 days in an ageing cohort of 20 uninoculated mice. This was associated with PrP plaque deposition in the anterior commissure (data not shown). We are currently investigating whether this pathology is transmissible on sub-passage.

### vCJD prions transmit to 117VV transgenic mice without producing florid plaques

We also investigated the pattern of neuropathology produced in vCJD-inoculated 117VV Tg30 mice. One of 3 positive samples showed abundant plaques in the cerebral cortex, hippocampus, thalamus and cerebellum (Figure 1M and O). Although neuronal loss was present, there were no florid plaques, consistent with the propagation of vCJD in the *PRNP* 129VV genotype [37], [40]. However, while only a few non-florid plaques are typically seen with vCJD transmission to the wild-type human PrP 129VV genotype [37], [40], the abundance of non-florid plaques associated with vCJD transmission to 117V mice is remarkable and clearly suggests a modifying effect of the mutation.

**Figure 4:**
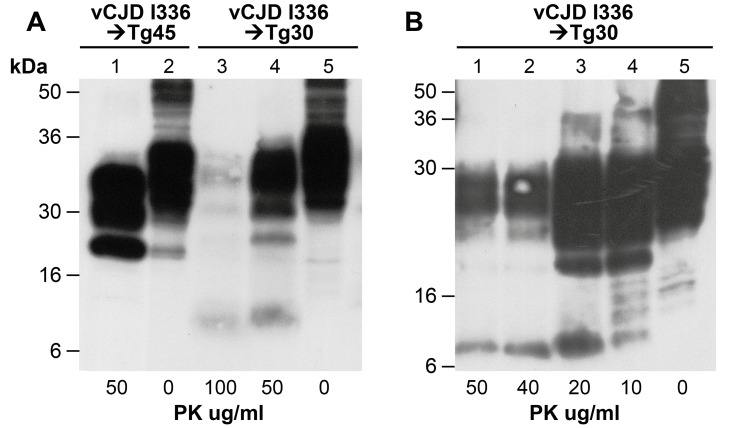
Immunoblot analysis of abnormal PrP propagated in the brains of 117VV Tg30 transgenic mice challenged with vCJD prions. All mice were inoculated with the same vCJD prion isolate. Immunoblots were analysed by enhanced chemiluminescence with monoclonal anti-PrP antibody ICSM 35. The provenance of the brain samples is designated above the lanes. (A) Lanes 3 and 4 show the predicted type 5 PrP^Sc^ bands seen when vCJD is propagated in transgenic mice expressing HuPrP with the codon 129VV genotype, compared with the detection of type 4 PrP^Sc^ in the brain of vCJD-inoculated 129MM Tg45 mouse (lanes 1 and 2). Type 5 shares the glycoform ratio of type 4 but differs in migrating more slowly on western blots because all 3 glycoform fragments of type 5 have higher apparent molecular masses than those of type 4. The lower signal intensity in lane 3 (100 µg/ml PK) compared to lane 4 (50 µg/ml PK) reflects PK-sensitivity of the vCJD-seeded 117V PrP^Sc^. The 8 kDa PrP fragments can be seen associated with only PK-digested prion-infected 117V PrP-expressing mouse brain samples. These truncated 8 kDa fragments are absent from either vCJD infected Tg45 mice (lanes 1 and 2) or vCJD-infected 117VV Tg30 brain samples not digested with PK (lane 5).These data confirm that the 8 kDa human PrP fragment is a disease-associated PrP degradation product. (B) Variable PK resistance in brain of 117VV Tg30 mouse inoculated with vCJD. The 8 kDa PrP fragment is only seen in PK digested lanes 1–4, but is absent in the same sample undigested with PK (lane 5).

Transmission of vCJD prions to transgenic mice homozygous for human PrP valine-129 invariably results in a strain shift from the characteristic type 4 PrP^Sc^ molecular signature to type 5 PrP^Sc^ [37], [40]. The presence of type 5 PrP^Sc^ fragment size in vCJD-inoculated 117VV Tg30 mouse brain (Figure 4A lane 4) compared to type 4 PrP^Sc^ propagated in the vCJD-inoculated 129MM Tg45 control brain (lane 1) clearly shows that the 117V mutation on the valine-129 allele does not influence the previously established strain shift phenomenon. Interestingly, a truncated PrP peptide of about 8 kDa that is associated with GSS A117V mutation was also seen on longer exposure in PK-digested (Figure 4A lanes 3 and 4) and PK-titrated samples (Figure 4B lanes 1 to 4). Notably, the 8 kDa fragment was not seen without PK digestion (lane 5 Figure 4A and 4B respectively), thus confirming this PrP fragment as disease specific. Indeed, in some vCJD-challenged 117V Tg30 mouse brains that were positive by immunohistochemistry, only the GSS-associated 8 kDa PrP fragment was detectable (Table 2).

## Discussion

We have demonstrated that GSS A117V is indeed a transmissible condition and properly designated an inherited prion disease rather than simply a prion proteinopathy without generation of prions. Additionally, we report that classical PrP^Sc^ is detectable in PrP 117V transgenic mouse brain using suitable conditions. The inability to detect classical PrP^Sc^ in patient brain had led to the proposal that the A117V mutation may cause pathology principally via an alternative pathway, namely through an increase in C-terminal transmembrane PrP, designated ^Ctm^PrP, to the total exclusion of PrP^Sc^ [8]. It has also not been shown whether or not 117V-PrP^C^ is convertible to PrP^Sc^. Using appropriate transgenic models challenged with classical CJD prion isolates, we have demonstrated that, despite the observed transmission barrier to clinical disease which can be explained by the 117V mutation producing a partial transmission barrier, 117V PrP^C^ is a competent substrate for conversion to PrP^Sc^. Notably, the newly generated PrP^Sc^ assumes the stable strain properties of the exogenous PrP^Sc^ and is therefore readily detectable on immunoblots.

Similarly, although transmission properties of GSS A117V prions in these mice were not typical of prion transmission to transgenic mice expressing the homotypic substrate, our detection of classical PrP^Sc^ is unprecedented and confirms that experimental conditions in our 117VV transgenic mice were favourable for replication of PrP^Sc^. However, in contrast to the stable PrP^Sc^ propagated from classical CJD prion transmission to these mice, the observation that PrP^Sc^ generated from GSS A117V prions *in vivo* was inherently unstable may in part explain the low clinical attack rates observed in the present study and the failure of previous transmission attempts. It is reasonable to infer that because the A117V-derived abnormal PrP is labile, prion replication and the probability of a sustained prion infection in these mice would have been greatly enhanced by the 2–3 fold over-expression of the substrate, 117V PrP^C^.

Given that the only protease-resistant PrP fragment found to-date in A117V patients' brains is the characteristic 8 kDa PrP fragment [26], our 117VV Tg30 line in which only 8 kDa PrP fragment was detectable has recapitulated the GSS A117V disease phenotype. Since the 8 kDa peptide was only seen as a proteinase-K resistant truncated fragment, it represents a GSS-specific PrP degradation product, the detection of which can be taken as a reliable surrogate marker for confirming prion disease in GSS A117V patients [26]. The possibility of classical PrP^Sc^ being present at low and undetectable levels in GSS A117V patient brain homogenates cannot be ruled out. It therefore remains to be determined whether the parent PrP conformer that generates the 8 kDa protease resistant PrP, is capable of initiating and sustaining prion infection or that transmissibility remains associated with classical PrP^Sc^ present below the threshold of detection. In this regard, even a successful serial passage of GSS A117V-challenged Tg30 mouse brains apparently propagating only the 8 kDa fragment and resulting in the propagation of classical PrP^Sc^, may not resolve this issue.

All previous reports of PrP point mutations causing spontaneous neurodegeneration have involved superimposing human PrP pathogenic mutations onto rodent PrP [7], [45], [46], and these studies have invariably reported very high incidences of spontaneous neurological dysfunction. Since destabilising effects measured in a mouse protein cannot be assumed to be equivalent in the human protein [47], [48], we have modelled the A117V mutation directly on human PrP. This difference in approach can explain the contrasting low incidence of spontaneous disease in our 117VV transgenic mice. The development of neurological dysfunction in transgenic mice expressing disease-associated mutations modelled on rodent PrP has been described as disease acceleration [49], because PrP^Sc^ has not been detectable and transmissibility has not been demonstrated conclusively. In this regard, transmissibility of spontaneous PrP plaque deposits in aged 117VV Tg30 mice is being investigated and will be reported in a subsequent publication.

The observation that vCJD prions transmit more readily, albeit subclinically, to 117VV Tg30 but not to 117VV Tg31 mice that have higher PrP expression levels was unexpected. However, whereas all vCJD-inoculated 117VV Tg31 mice had a maximum post-inoculation survival period of 547 days (culled in the range 292–547 days), 6/7 117VV Tg30 mice challenged with the same inoculum survived in the range of 627–811 days post-inoculation. These data suggest that very prolonged replication periods may be required for pathological PrP to become detectable in vCJD-challenged 117V transgenic mice by either IHC or immunoblotting. Subpassage of apparently negative brains could be used to explore this, however this is not a central part of this study.

Our results may have wider implications for other inherited prion diseases that have not been shown to be transmissible as yet. Firstly, it is possible that demonstration of transmissibility of such inherited prion diseases would require specific transgenic models with over-expression of the relevant mutant human PrP, rather than endogenous levels of mutant PrP expression, if transmissibility is to be demonstrated within the lifespan of a mouse. The transient detection of PrP^Sc^ in our study suggests that A117V-associated PrP^Sc^ is labile and readily susceptible to proteases. This results in progressive reduction of PrP^Sc^ to undetectable, yet still potentially infectious levels. In this regard, failure to detect low levels of PrP^Sc^ in the past from patient brain samples could be due to technical limitations of currently available biochemical techniques, rather than its absence.

## Methods

### Ethics statement

Storage and biochemical analysis of human tissue samples and transmission studies to mice were performed with written informed consent from patients or relatives under approval from the Local Research Ethics Committee of UCL Institute of Neurology/National Hospital for Neurology and Neurosurgery and the code of practice specified in the Human Tissue Authority licence held by UCL Institute of Neurology. Work with mice was performed under licence granted by the UK Home Office (Animals (Scientific Procedures) Act 1986 ; Project Licence number 70/6454) and conformed to University College London institutional and ARRIVE guidelines.

### Generation of transgenic mice

The 759 bp human PrP ORF was amplified by PCR with pfu polymerase from genomic DNA prepared from the brain of a patient with the inherited prion disease A117V mutation, using forward primer 5′-GTCGACCAGTCATTATGGCGAACCTT-3′ and reverse primer 5′-CTCGAGAAGACCTTCCTCATCCCACT-3′. Restriction sites Sal I and XhoI (underlined) were introduced in the forward and reverse primers respectively for cloning. The sequence was confirmed and ligated into the cosmid vector CosSHaTet [33]. Microinjection of the purified DNA was carried out according to standard protocol into single cell eggs of *Prnp* null mice [38] which had been backcrossed onto an FVB/N background. Genotyping was performed by PCR and PrP expression levels estimated by Western blot analysis as previously reported [50]. Two homozygous lines were established for HuPrP 117V described as Tg(HuPrP^117V,129V+/+^ *Prnp^o/o^*)-30 (designated human PrP 117VV Tg30) and Tg(HuPrP^117V,129V+/+^ *Prnp^o/o^*)-31 (human PrP 117VV Tg31) with mutant transgene expression levels of 2 and 3 times respectively, compared to pooled normal human brain levels.

### Transmission studies

Strict bio-safety protocols were followed. Inocula were prepared, using disposable equipment for each inoculum, in a microbiological containment level 3 laboratory and inoculations performed within a class 1 microbiological safety cabinet. Ten mice per group of 117VV Tg31 transgenic mice were inoculated with prion isolates comprising human brain homogenates from: three separate IPD A117V cases; two sporadic CJD cases; three iatrogenic CJD cases; one case of vCJD and one mouse brain isolate from vCJD passaged once in Tg152 mice expressing wild-type human PrP V129 (containing type 5 PrP^Sc^) [37], [40], as detailed in Table 1. Similarly, the second 117VV transgenic line, 117VV Tg30 mice were challenged with the same three IPD A117V inocula, and 1 inoculum each of iatrogenic CJD and vCJD as detailed in Table 2. All cases were neuropathologically confirmed.

The genotype of each mouse was confirmed by PCR of tail DNA prior to inclusion and all mice were uniquely identified by sub-cutaneous transponders. Disposable cages were used and all cage lids and water bottles were also uniquely identified by transponder and remained with each cage of mice throughout the incubation period. Mice were anaesthetised with a mixture of halothane and O_2_, and intracerebrally inoculated into the right parietal lobe with 30 µl of a 1% brain homogenate prepared in phosphate-buffered saline (PBS). All mice were thereafter examined daily for clinical signs of prion disease. Mice were killed if they exhibited any signs of distress or once a diagnosis of prion disease was established.

### Neuropathology and immunohistochemistry

Mice were culled by CO_2_ asphyxiation. Brain was fixed in 10% buffered formol saline and then immersed in 98% formic acid for 1 hour and paraffin wax embedded. Serial sections of 4 µm thickness were pre-treated by boiling for 10 min in a low ionic strength buffer (2.1 mM Tris, 1.3 mM EDTA, 1.1 mM sodium citrate, pH 7.8) before exposure to 98% formic acid for 5 min. Abnormal PrP accumulation was examined using anti-PrP monoclonal antibody ICSM 35 (D-Gen Ltd, London) on a Ventana automated immunohistochemical staining machine (Ventana Medical Systems Inc., Tucson, Arizona) using proprietary secondary detection reagents (Ventana Medical Systems Inc) before development with 3′3 diaminobenzedine tetrachloride as the chromogen. Harris haematoxylin and eosin staining was done by conventional methods. Appropriate controls were used throughout.

### Western blotting

Preparation of brain homogenates (10% w/v in phosphate buffered saline), proteinase K digestion (titration up to 100 µg/ml for 1 h at 37°C), and subsequent western blotting was performed as described previously [51]. For primary screening of both transgenic and wild type mouse brain homogenates, blots were probed with either a monoclonal antibody which detects human, but not mouse, PrP (3F4 ([52])) or a biotinylated anti-PrP monoclonal antibody which recognises both human and mouse PrP (biotinylated-ICSM 35 (D-Gen Limited, London)) in conjunction with an avidin-biotin-alkaline phosphatase conjugate (Dako) and development in chemiluminescent substrate (CDP-Star; Tropix Inc). Primary screening of brain homogenates was performed blind to sample identity.

## Supporting Information

Figure S1 **H&E staining showing semi-quantitative scale used in scoring variable neuronal loss in the hippocampus of A117V-inoculated mice and PrP plaques in brain of a PBS-inoculated 117VV Tg31 mouse.** (A) Coronal section of the anterior commissure on level Bregma +2 mm showing PrP deposits in a PBS-inoculated Tg31 mouse. a, location of PrP deposits within and surrounding the anterior commissure (ac). Other structures seen on this level are the piriform cortex (Pir), the anterior olfactory nucleus, medial part (AOM) and the forceps minor of the corpus callosum (fmi). b, high power magnification of the anterior commissure shows multiple small plaques within the white matter and immediately adjacent to it. Note the spongiform changes in the anterior commissure. Scale bar = 1200 µm (A) and 250 µm (B). (B) Upper row (a, c, e, g) shows progressive thinning of the neuronal layer of the hippocampus. Lower row (b, d, f, h) shows close up of the neuronal layer in each corresponding figure above, with blue arrows highlighting drop outs of neurones. Definition of values for neuronal loss: 0: No neuronal loss; +: Drop out of single neurones either focally or within the Ammon's horn (AH), leaving the AH continuity intact; ++: Focal or regional drop out, interrupting the continuity of the AH and creating a small-medium gap (up to 1/3 of the length of the AH); +++: Neuronal drop out leaving gaps of more than 1/3 of the AH's length. Scale bar = 500 µm for top panel and 125 µm for the bottom panel.
(TIF)

Figure S2 **Immunoblot analyses of the brains of 117VV Tg31 mice challenged with GSS A117V prions showing time-course of degradation due to freeze-thawing.** (A) Samples initially digested at 100 µg/ml PK at 37°C for 1 hour (lanes 3–8) showed variable digestion but the 8 kDa fragment was already visible (lanes 3 and 4). (B) Repeat immunoblotting performed on the same samples after 1 freeze-thaw, using the same PK digestion conditions, confirmed the 8 kDa fragment as the main detectable PrP fragment in brains of A117V-challenged Tg31 mice (lanes 3 and 4). All other fragments are almost completely degraded (lanes 3–8). The positive control used in lanes 1 and 2 of both panels A and B was from a transgenic mouse expressing wild type HuPrP-129MV challenged with sporadic CJD inoculum.
(TIF)
