# Supplementary figures and images for: Inherited Prion Disease A117V Is Not Simply a Proteinopathy but Produces Prions Transmissible to Transgenic Mice Expressing Homologous Prion Protein
Source: PLoS Pathog. 2013 Sep 26;9(9):e1003643. doi: 10.1371/journal.ppat.1003643 (PMC3784465; doi:10.1371/journal.ppat.1003643)

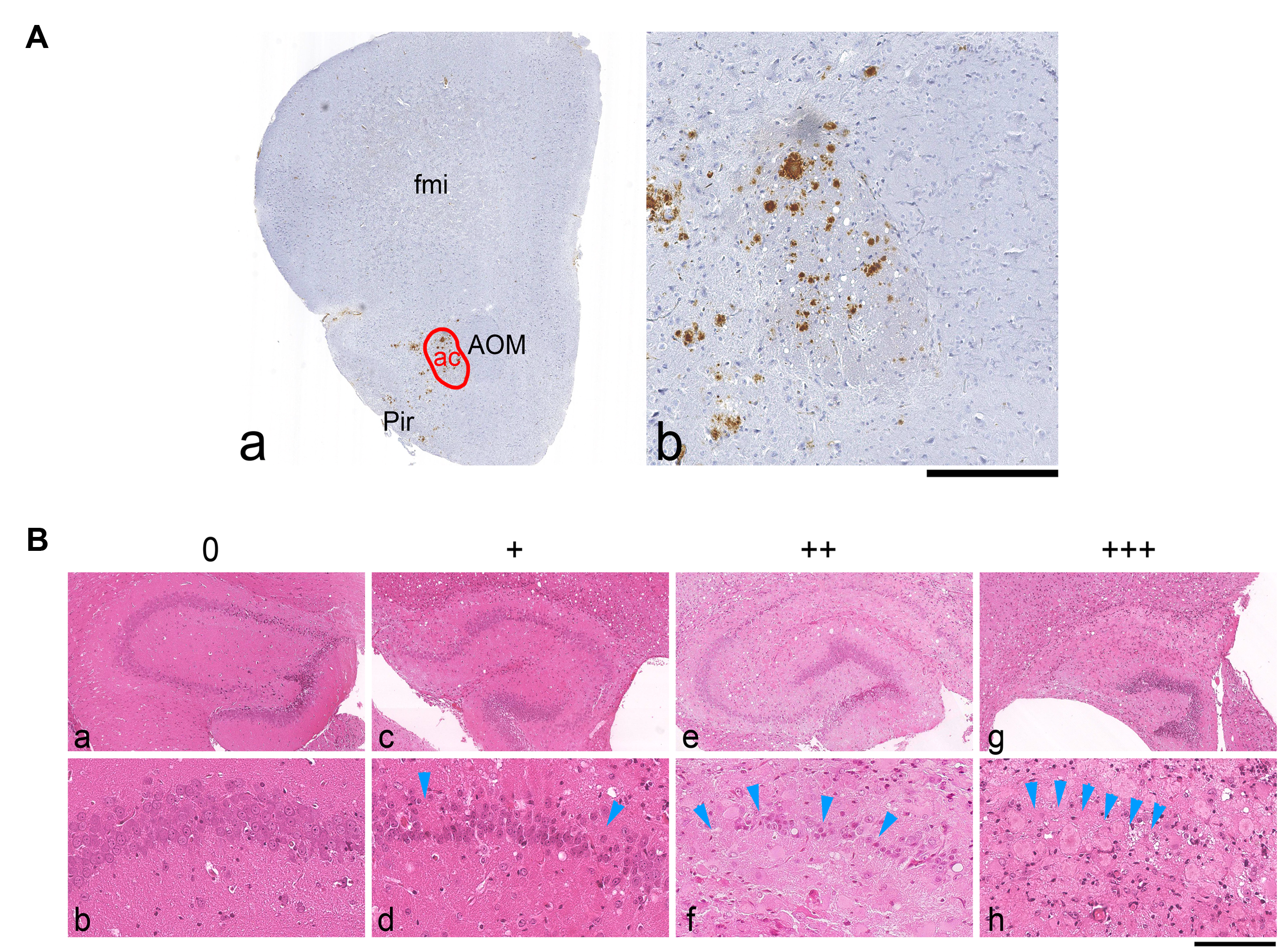

Supplement: Figure S1 — H&E staining showing semi-quantitative scale used in scoring variable neuronal loss in the hippocampus of A117V-inoculated mice and PrP plaques in brain of a PBS-inoculated 117VV Tg31 mouse. (A) Coronal section of the anterior commissure on level Bregma +2 mm showing PrP deposits in a PBS-inoculated Tg31 mouse. a, location of PrP deposits within and surrounding the anterior commissure (ac). Other structures seen on this level are the piriform cortex (Pir), the anterior olfactory nucleus, medial part (AOM) and the forceps minor of the corpus callosum (fmi). b, high power magnification of the anterior commissure shows multiple small plaques within the white matter and immediately adjacent to it. Note the spongiform changes in the anterior commissure. Scale bar = 1200 µm (A) and 250 µm (B). (B) Upper row (a, c, e, g) shows progressive thinning of the neuronal layer of the hippocampus. Lower row (b, d, f, h) shows close up of the neuronal layer in each corresponding figure above, with blue arrows highlighting drop outs of neurones. Definition of values for neuronal loss: 0: No neuronal loss; +: Drop out of single neurones either focally or within the Ammon's horn (AH), leaving the AH continuity intact; ++: Focal or regional drop out, interrupting the continuity of the AH and creating a small-medium gap (up to 1/3 of the length of the AH); +++: Neuronal drop out leaving gaps of more than 1/3 of the AH's length. Scale bar = 500 µm for top panel and 125 µm for the bottom panel. (TIF) [file ppat.1003643.s001.tif]

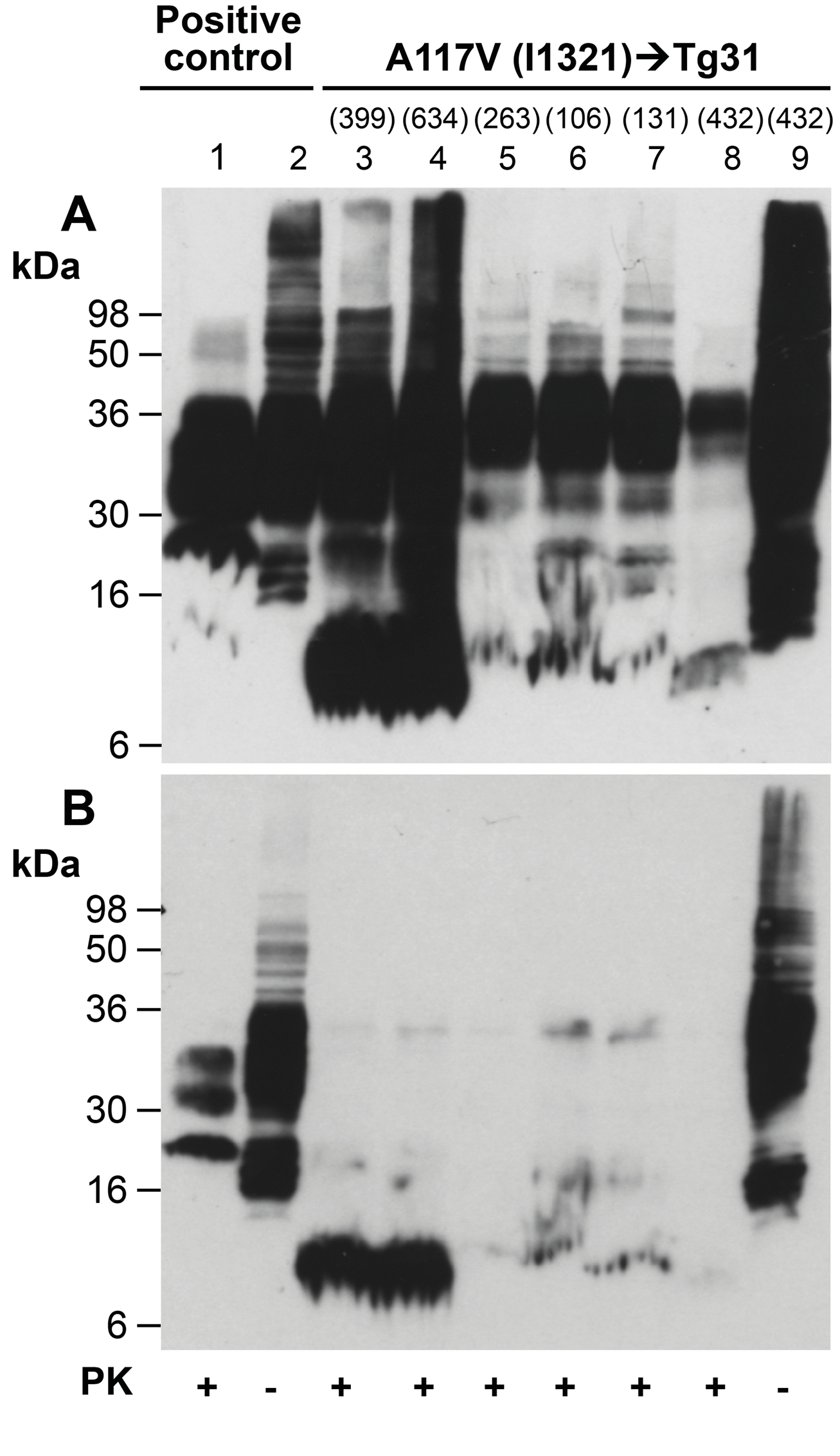

Supplement: Figure S2 — Immunoblot analyses of the brains of 117VV Tg31 mice challenged with GSS A117V prions showing time-course of degradation due to freeze-thawing. (A) Samples initially digested at 100 µg/ml PK at 37°C for 1 hour (lanes 3–8) showed variable digestion but the 8 kDa fragment was already visible (lanes 3 and 4). (B) Repeat immunoblotting performed on the same samples after 1 freeze-thaw, using the same PK digestion conditions, confirmed the 8 kDa fragment as the main detectable PrP fragment in brains of A117V-challenged Tg31 mice (lanes 3 and 4). All other fragments are almost completely degraded (lanes 3–8). The positive control used in lanes 1 and 2 of both panels A and B was from a transgenic mouse expressing wild type HuPrP-129MV challenged with sporadic CJD inoculum. (TIF) [file ppat.1003643.s002.tif]
